# Safety and efficacy of autologous adipose tissue-derived stem cell transplantation in aging-related low-grade inflammation patients: a single-group, open-label, phase I clinical trial

**DOI:** 10.1186/s13063-024-08128-3

**Published:** 2024-05-08

**Authors:** Ngoc-Huynh Ton Nguyen, Hao Thanh Phan, Phong Minh Le, Lan-Huong Thi Nguyen, Thuy Thi Do, Thien-Phuc Thanh Phan, Trinh Van Le, Thanh Minh Dang, Chinh-Nhan Lu Phan, Tung-Loan Thi Dang, Nhung Hai Truong

**Affiliations:** 1 DNA International General Hospital, Ho Chi Minh City, 700000 Vietnam; 2 Laboratory of Stem Cell Research and Application, University of Science, VNU HCM, Ho Chi Minh City, 700000 Vietnam; 3 Stem Cell Institute, University of Science, VNU HCM, Ho Chi Minh City, 700000 Vietnam; 4 Faculty of Biology and Biotechnology, University of Science, VNU HCM, Ho Chi Minh City, 700000 Vietnam; 5 Viet Nam National University, Ho Chi Minh City, 700000 Vietnam

**Keywords:** Mesenchymal stem cell, Immune modulation, Inflammatory, Proinflammatory, Inflamm-aging

## Abstract

**Background:**

Inflamm-aging is associated with the rate of aging and is significantly related to diseases such as Alzheimer’s disease, Parkinson’s disease, atherosclerosis, heart disease, and age-related degenerative diseases such as type II diabetes and osteoporosis. This study aims to evaluate the safety and efficiency of autologous adipose tissue-derived mesenchymal stem cell (AD-MSC) transplantation in aging-related low-grade inflammation patients.

**Methods:**

This study is a single-group, open-label, phase I clinical trial in which patients treated with 2 infusions (100 million cells i.v) of autologous AD-MSCs were initially evaluated in 12 inflamm-aging patients who concurrently had highly proinflammatory cytokines and 2 of the following 3 diseases: diabetes, dyslipidemia, and obesity. The treatment effects were evaluated based on plasma cytokines.

**Results:**

During the study’s follow-up period, no adverse effects were observed in AD-MSC injection patients. Compared to baseline (D-44), the inflammatory cytokines IL-1α, IL-1β, IL-8, IL-6, and TNF-α were significantly reduced after 180 days (D180) of MSC infusion. IL-4/IL-10 at 90 days (D90) and IL-2/IL-10 at D180 increased, reversing the imbalance between proinflammatory and inflammatory ratios in the patients.

**Conclusion:**

AD-MSCs represent a potential intervention to prevent age-related inflammation in patients.

**Trial registration:**

ClinicalTrials.gov number is NCT05827757, first registered on 13th Oct 2020

**Supplementary Information:**

The online version contains supplementary material available at 10.1186/s13063-024-08128-3.

## Introduction

The term “inflammatory aging” or “inflamm-aging” was first mentioned by Franceschi et al. in 2000 [1], representing a new direction of research on aging and aging-related diseases. Castellani GC et al. formulated the biomedical hypothesis of aging [2], suggesting that chronic, low-grade inflammation is a driver of the inflammatory pathological process and is associated with aging. Accordingly, “inflammatory aging” was defined as a chronic inflammatory state with age-related increases in serum proinflammatory cytokines [1, 3, 4]. According to the publication of Chen et al. [5], to date, no specific clinical cutoff value for inflammatory aging has been published, so most of the studies have established that the average cytokine values vary based on the characteristics of the study population and the modalities of cytokine measurement. Generally, values > 95% percentile of normal healthy individuals are considered pathological. The dynamic balance of the network of proinflammatory and anti-inflammatory cytokines normally maintains the physiological function of inflammation in the body. Losing the balance between anti-inflammatory and proinflammatory states can lead to pathological changes. Many authors have suggested that inflammation that persists during inflammatory aging is the cause of inflammation-related diseases [6, 7].

Inflammatory aging has been identified as a significant risk factor for morbidity and mortality in older people [8]. Inflammatory aging is a determinant of the rate of aging and longevity. It is strongly associated with diseases such as Alzheimer’s disease, Parkinson’s disease, acute sclerosis, multiple sclerosis, atherosclerosis, heart disease, age-related degenerative diseases [4], type II diabetes [9], osteoporosis and insulin resistance [10], cancer, and others [11]. Research on inflammatory aging is still in its early stages; the mechanisms, biomarkers, research models, and inflammatory aging interventions still need to be fully elucidated. Approaching to control age-related inflammation is expected to reduce human disease.

Mesenchymal stem cells (MSCs) have been researched, developed, and applied in many fields. The data published by the US National Institutes of Health on the online portal ClinicalTrials.gov show approximately 8000 clinical studies on MSCs in various diseases (an update to 1/7/2019). In particular, many clinical studies have shown the therapeutic effect of MSCs on many diseases related to inflammation, such as rheumatoid arthritis (RA) and lupus erythematosus [12, 13]. MSCs are remarkably effective in controlling the inflammatory cytokines that are elevated in rheumatoid arthritis [14], including tumor necrosis factor-alpha (TNFα) [15], which is a cytokine that plays a significant role in pathogenesis. In addition to reducing inflammation in RA patients, MSCs act on and neutralize TNFα with human antibodies and minimize disease progression [16, 17]. MSCs have also been shown to effectively control inflammation in multiple sclerosis (MS), a chronic autoimmune disease of the central nervous system usually diagnosed in adults [18]. The mechanism of MSC treatment modulates the immune response [19, 20] and reduces the expression of proinflammatory cytokines, which is shifting the immune response from predominantly proinflammatory T helper cell 1 to the anti-inflammatory T helper cell 2. Although MSCs have been applied in treating many specific diseases, there are few clinical trial studies on MSCs and their effects on inflammatory aging. In this study, in addition to evaluating the safety of adipose tissue-derived MSC transplantation therapy, we assessed the effects of this therapy on proinflammatory cytokines: interleukin (IL-1α/β), TNF-α, IL-2, IL-6, IL-8, vascular endothelial growth factor (VEGF), and interferon (IFN-γ), and the anti-inflammatory cytokines: IL-4, IL-10, monocyte chemoattractant protein (MCP-1), and epidermal growth factor (EGF) in Vietnamese patients with comorbidities. This study aims to contribute to a common understanding of inflammatory aging and aging-related low-grade inflammation, helping control diseases related to the body’s aging process.

## Materials and methods

### Ethical declaration

This study was conducted in accordance with the ethical principles contained in the Declaration of Helsinki, the Good Clinical Practice Guidelines (ICH GCP E6R2), the Good Clinical Practice guidelines of the Vietnamese Ministry of Health, and current regulatory requirements and policies of DNA International General Hospital (Ho Chi Minh City Vietnam) on ethics in biomedical research. The study on participants was approved by the Ethics Committee of DNA International General Hospital (Ho Chi Minh City 700,000, Vietnam) for biomedical research (decision number was No.21/CN-HĐĐĐ), the National Ethics Committee (decision no. 13/CN-HĐĐĐ), and the Vietnam Ministry of Health (1690/QĐ-BYT). Before participation, all patients provided written informed consent after receiving adequate information. This research has been registered with ClinicalTrials.gov (NCT05827757).

### Patient recruitment

The current trial is a phase I single-group, open-label, controlled before-after clinical trial to evaluate the safety and efficacy of autologous AD-MSC transplantation on proinflammatory cytokines and anti-inflammatory cytokines in aging-related low-grade inflammation patients. The study was conducted at DNA International General Hospital (Ho Chi Minh City, Vietnam). The participant enrollment took place from December 2020 to October 2022. Study subjects who meet the inclusion and do not meet the exclusion criteria of the study will be recruited with a minimum sample size of 12 patients (the minimum number of patients required by the Ministry of Health of Vietnam for the phase I trial).

The following are the inclusion criteria: (1) males or females aged 40–64 years; (2) TNF-α index > 11 pg/ml and IL6 index > 1.23 pg/ml (blood samples were tested on the multiplex system at Cho Ray Hospital, Ho Chi Minh City, Vietnam); (3) possesion of at least two of the following three comorbidities: diabetes, dyslipidemia, and obesity (the diagnosis of the comorbidities was made according to the Ministry of Health’s general guidelines); (4) stable use of medications for the previous 3 months to treat the previously mentioned comorbidities (diabetes, dyslipidemia, and obesity); and (5) agreement to participate in the study and to comply with the research examination and evaluation process.

The following are the exclusion criteria: (1) patients with coagulopathy, (2) history of or current severe heart failure, (3) acute respiratory disease at the time of screening, (4) patients with cancer or other acute illness requiring treatment, (5) history of allergy to anesthetics and antibiotics, (6) currently/planning to participate in another clinical trial during the study period, and (7) possessing additional conditions or circumstances that make it difficult to provide treatment, according to the researcher. Pathology of disease in the exclusion criteria was defined according to the Guidelines of the Ministry of Health of Vietnam.

### Patient follow-up and treatment

During the trial, patients with diabetes, dyslipidemia, and obesity continued to take their medications. While adjusting the drug dose limited to the maximum dosage, any changes to the dose were used simultaneously with the experiment treatment and were consulted by a doctor to ensure patient safety.

**Fig. 1:**
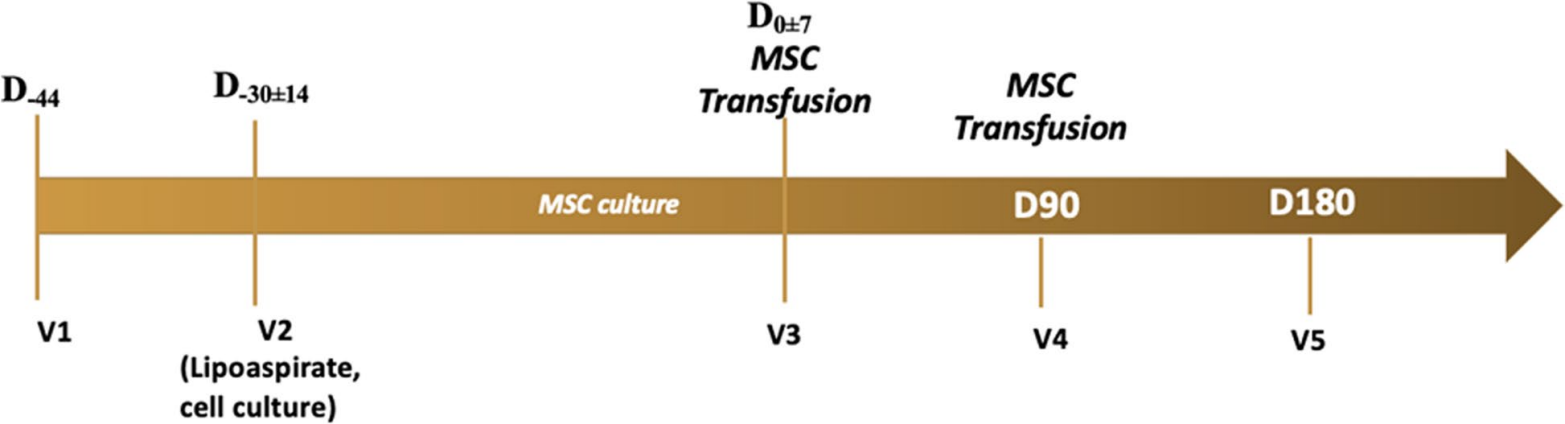
Study time points

Patients in the study underwent a total of 5 evaluation visits: visit to participate in the study (V1, D-44 ± 7), visit to harvest and culture cells (V2, D-30 ± 14), first MSC transplant (V3, D0), second MSC transplant (V4, D90 ± 7), and last visit/end of study visit (V5, D180 ± 14). In V1, patients were screened and selected for study. In V2, selected patients were liposuctioned, and their cells were cultured. First, 100 million autologous AD-MSC were i.v transfused in V3, followed by a second transfusion in V4. The monitoring and evaluation were performed in V5 (Fig. 1).

### MSC preparation and administration

Autologous MSCs from adipose tissue were harvested, cultured, cryopreserved, and quality checked according to the institute’s standard operating procedure (DNA International General Hospital Joint Stock Company, Ho Chi Minh City, Vietnam). Briefly, MSCs were isolated from the patient’s adipose tissue after lipoaspiration by collagenase (code N0002778, AMSBIO, MA, USA). MSCs were expanded in an MSC culture medium kit (ADSCCult I, Stem Cell Institute, Vietnam) to passage 2 or 3 and then frozen in liquid nitrogen for later infusion. Prior to storage or transfusion, the MSCs were subjected to quality control tests as described in Table 1, including mycoplasma (MycoAlert™ PLUS, code: LT07-703, Lonza, Switzerland), sterility (Fluid Thioglycollate Medium and Soybean-Casein Digest Medium, Merck Millipore, Germany), endotoxin < 0.5 EU/ml (Kinetic-QCL™ Kinetic Chromogenic LAL Assay, code: 50-650U, Lonza), cell survival > 90% (Trypan Blue staining count), and marker expression for MSC identification [21] (BD Stemflow™ Human MSC Analysis Kit, code: 562245, BD Biosciences, NJ, USA). All patients were given 2 intravenous autologous infusions on day 0 (D0) and day 90 (D90). A total of 100 million single cells were suspended in 0.9% sterile saline solution and were given intravenously at a 5-ml/min rate for 45 min. The safety variables were monitored for the whole duration of the study.

**Table 1:** Adipose tissue-derived mesenchymal stem cell quality

**Patient ID**	**Mycoplasma**	**Bacteria**	**Endotoxin**	**Cell survival (%)**	**Cell number (× 10**^**6**^**)**	**Marker (%)**				
						**CD44**	**CD73**	**CD90**	**CD45/14/CD19**	**HLA-DR**
01-01-P1031	Neg	No	< 0.5 EU/ml	96	107	99	93	100	2.7	2.7
01-01-P1034	Neg	No	< 0.5 EU/ml	97	110	99.4	97	99.8	2.4	2.4
01-01-P1035	Neg	No	< 0.5 EU/ml	96	116.7	99.7	94	98.9	3.1	3.1
01-01-P1038	Neg	No	< 0.5 EU/ml	96	107.8	98.2	91.7	99.9	2.1	2.1
01-01-P1039	Neg	No	< 0.5 EU/ml	96	120	98.5	94.3	99.4	2.6	2.6
01-01-P1041	Neg	No	< 0.5 EU/ml	98	112	98.9	97.1	99.2	2	2
01-01-P1042	Neg	No	< 0.5 EU/ml	93	115	99.9	93.5	100	0.9	0.9
01-01-P1043	Neg	No	< 0.5 EU/ml	93	111.5	98.5	99.2	100	3.8	3.8
01-01-P1044	Neg	No	< 0.5 EU/ml	98	120	99.3	93.2	98.9	3.1	3.1
01-01-P1045	Neg	No	< 0.5 EU/ml	99	111	99	91.4	99.5	2.5	2.5
01-01-P1046	Neg	No	< 0.5 EU/ml	96	117	99.9	94.4	99.9	0.4	0.4
01-01-P1047	Neg	No	< 0.5 EU/ml	97	107.2	97	92	99.6	2.9	2.9
**Mean**	**Neg**	**No**	**< 0.5 EU/ml**	**96.25**	**112.93**	**98.94**	**94.23**	**99.59**	**2.38**	**2.38**
**SD**	**Neg**	**No**	**< 0.5 EU/ml**	**1.82**	**4.72**	**0.82**	**2.40**	**0.41**	**0.94**	**0.94**

### Outcomes

#### Safety

Safety assessments included adverse events (AEs) and serious adverse events (SAEs) during the entire study follow-up period. AEs are also classified separately, including patients who stopped or withdrew from the study. Frequency of AEs and serious SAEs within 24 h after stem cell transplant was measured. This study used the National Institutes of Health’s (NIH) AE/SAE assessment and recording guideline document, Common Terminology Criteria for Adverse Events CTCAE version 5.0, which accompanies the study.

Clinical tests used to evaluate the safety include basic hematologic and biochemical tests performed at a local laboratory. Changes in vital signs and physical examination over time were not analyzed separately from the study protocol. In cases where these changes met the criteria of an AE/SAE, the changes were analyzed jointly with the AE/SAEs.

#### Efficiency

##### Cytokine measurements

The serum concentrations of IL-1α/β, TNF − α, IL-2, IL-6, IL-8, VEGF, IFN-γ, IL-4, IL-10, MCP-1, and EGF were measured by a cytokine growth factor array kit (cat EV3513, Randox, UK) at Cho Ray Hospital, Ho Chi Minh City, Vietnam.

Absolute changes in proinflammatory cytokines (IL-1α/β, TNF-α, IL-2, IL-6, IL-8, VEGF, IFN-γ) and anti-inflammatory cytokines (IL-4, IL-10, MCP-1, EGF) after transplantation of AD-MSC at D90 and D180 were compared to the time before study D-44.

Ratio of proinflammatory cytokines and anti-inflammatory cytokines after treatment at D90 and D180 was compared with pretreatment levels (IL-4/IL-10, IL-1β/IL-10, IL-6/IL-10, IL-2/IL-10, IL-6/IL-10, IL-1β/EGF).

##### Statistical analysis

The study screened 21 patients and included 12 patients in the study. No imputation for missing variables was performed since the data set was complete for the 12 patients before research and at day 90 and day 180 that included the measurement of all 12 cytokines.

Continuous data are presented with descriptive statistics (e.g., mean and standard deviation, median, range, Q1, Q3). The 95% CI of a mean, a ratio, or the difference between two means or two ratios will be calculated as appropriate. The charts show the average value of the cytokines with their standard error (SE) and visualized by the GraphPad Software.

The Kolmogorov-Smirnov and Shapiro-Wilks tests determined whether the data followed a normal distribution. In this study, Fisher’s exact test was used to compare the two ratios. With quantitative testing values, according to the nonstandard distribution assumption, the nonparametric Wilcoxon signed-rank test was used for comparison. Differences were considered significant at *p* < 0.05 unless otherwise stated.

The statistical analyses were performed with the SAS® System 9.4 software (SAS Institute Inc., Cary, NC).

## Results

### Patients population

In total, 21 patients were recruited between December 2020 and October 2022, with 12 selected for the study. The screening process resulted in the exclusion of 7 patients who did not meet the study criteria; the remaining 2 patients were rejected from continuing with the study protocol. The demographic characteristics of the patients in the study are presented in Table 2 (full analysis set, including 12 patients). Of the 12 patients, there are 6 patients with diabetes, dyslipidemia, and obesity; 4 patients with diabetes and dyslipidemia; 1 patient with dyslipidemia and obesity; and 1 patient with diabetes and obesity.

**Table 2:** Demographic characteristics of the study subject

	**Gender**		**Total**
	**Male**	**Female**	
**Age**			
***n***	7	5	12
**Mean (SD)**	53.0 (7.1)	48.8 (8.4)	51.3 (7.6)
**Weight (kg)**			
***n***	7	5	12
**Mean (SD)**	75.7 (16.6)	56.9 (10.4)	67.9 (16.8)
**BMI**			
***n***	7	5	12
**Mean (SD)**	28.50 (4.87)	23.65 (4.09)	26.48 (5.03)

In summary, seven of the patients were male, five were female, and their average age ranged from 40 to 63 years. The average weight of the patients was 67.9 (16.8) kg, and their BMI was 26.48 (5.03) kg/cm^2^. All participants were Kinh ethnic patients.

### The safety of the study protocol in patients

First, the safety monitoring of the patients is presented in Table 3, and the details of events are in the Supplement table. In summary, during the duration of the study, 75% of patients experienced adverse reactions such as tuberculosis infection, white blood cell count increase, cortisol decrease, and dry eyes. The majority of AEs in patients (66.77%) were unrelated to the study intervention. In a study involving a total of 12 patients, 1 patient (8.3%) experienced a possible AEs related to stem cell intervention which was a sign of fatigue. According to NAS analysis, these AEs were mild, ensuring that the study protocol is safe for these patients.

**Table 3:** The safety of patient intervention

**Adverse events**	**Men (*n* = 7)**	**Women (*n* = 5)**	**Total (*n* = 12)**
All events, *n* (%) [95% CI]	5 (71.43%) [27.17–96.33%]	4 (80.00%) [24.82–99.49%]	9 (75.00%) [41.13–94.51%]
Nonrelation, *n* (%) [95% CI]	5 (71.43%) [27.17–96.33%]	3 (60.00%) [13.84–94.73%]	8 (66.67%) [33.86–90.08%]
Relation, *n* (%) [95% CI]		1 (20.00%) [0.51–71.64%]	1 ( 8.33%) [0.21–38.48%]
Mild, *n* (%) [95% CI]	5 (71.43%) [27.17–96.33%]	4 (80.00%) [24.82–99.49%]	9 (75.00%) [41.13–94.51%]

### Effectiveness of stem cell transplantation on inflammation cytokines

**Fig. 2:**
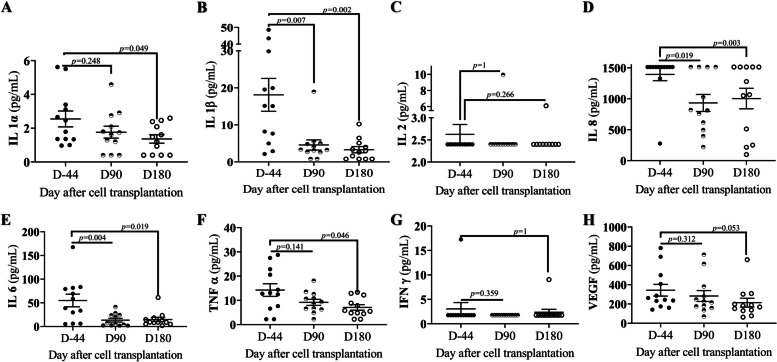
The effect of adipose stem cell transplantation on inflammatory cytokines in patients. Baseline (D-44), 90 days (D90), and 180 days (D180) after stem cell transplant; the patient’s inflammatory cytokines level were compared to their D-44 at D90 and D180 after transplant. **A** Interleukin 1α. **B** Interleukin 1β. **C** Interleukin 2. **D** Interleukin 8. **E** Interleukin 6. **F** Tumor necrosis factor α. **G** Interferon γ. **H** Vascular endothelial growth factor. The Wilcoxon signed-rank test was used to compare D90 and D180 with the D-44, and a *p*-value < 0.05 was considered statistically significant

The effect of stem cell transplantation on inflammatory cytokines in patients was measured at baseline (D-44), D90, and D180 after the first cell transplantation (Fig. 2). Specifically, D90 after stem cell transplantation, IL-1β (*p* = 0.007, Fig. 2B), IL-8 (*p* = 0.018, Fig. 2D), and IL-6 (*p* = 0.0035, Fig. 2D) inflammatory cytokines were statistical significantly reduced compared to baseline. Until D180, the inflammatory cytokines IL-1α (*p* = 0.04, Fig. 2A), IL-1β (*p* = 0.003, Fig. 2B), IL-8 (*p* = 0.03), IL-6 (*p* = 0.01, Fig. 2E), and TNF-α (*p* = 0.04, Fig. 2D) decreased. There was no statictical significant effect of stem cell transplantation on IL-2, IFN-γ, or VEGF (Fig. 2C, G, H). Therefore, stem cell transplantation has a potent anti-inflammatory effect on patients for up to D180 following infusion.

### The effectiveness of stem cell transplantation with regard to anti-inflammatory cytokines

**Fig. 3:**
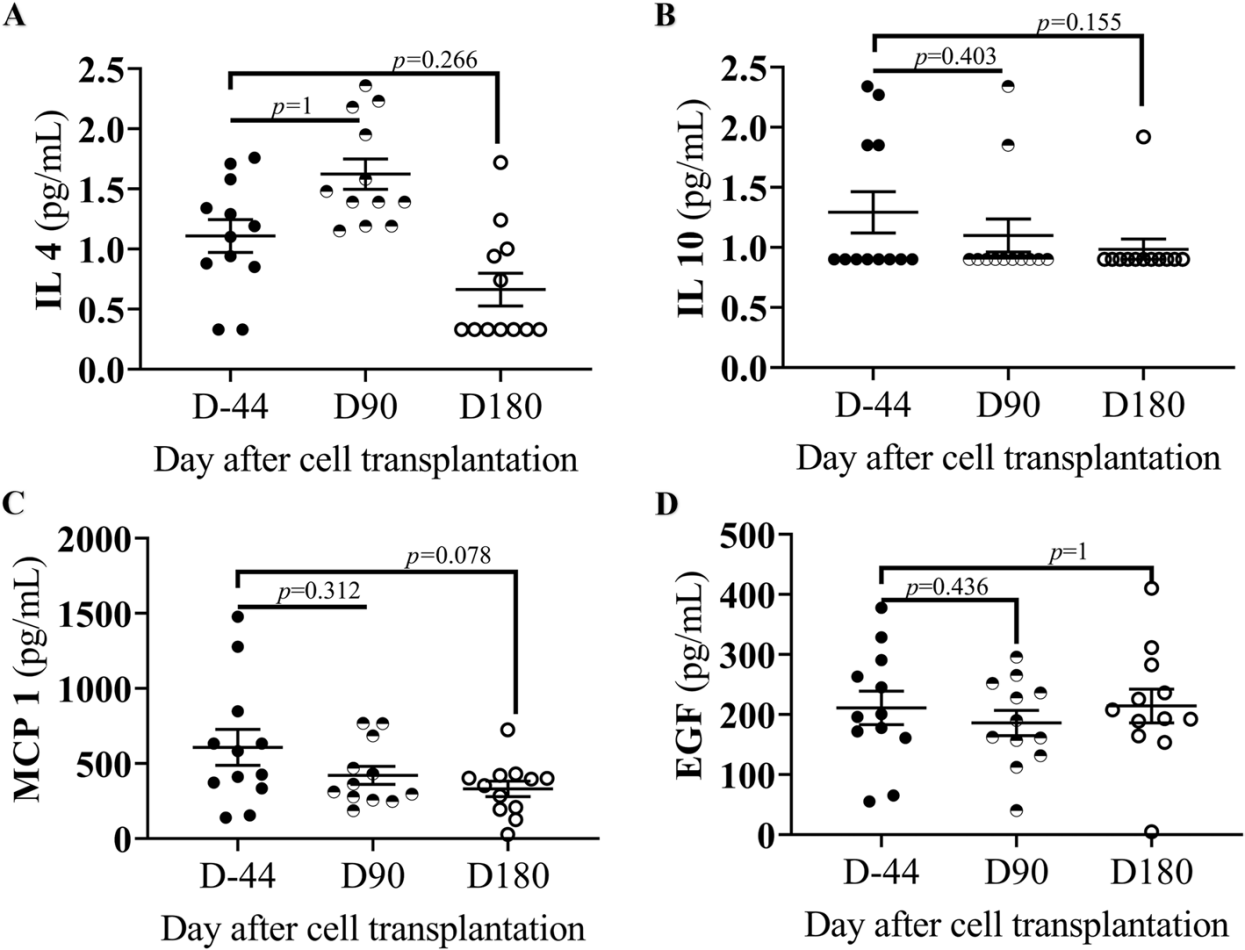
Anti-inflammatory cytokine effects of adipose stem cell transplantation in patients. The patients’ anti-inflammatory cytokines at D-44, D90, and D180 after stem cell transplant. **A** Interleukin 4. **B** Interleukin 10. **C** Monocyte chemoattractant protein-1. **D** Epidermal growth factor. The Wilcoxon signed-rank test was used to compare D90 and D180 with the baseline (D-44), and a *p*-value < 0.05 was considered statistically significant

In contrast to the proinflammatory cytokine, the anti-inflammatory cytokines IL-4, IL-10, MCP-1, and EGF did not have a statistically significant effect on stem cell transplantation in patients (Fig. 3). The concentrations of IL-4 at D-44, D90 after transplantation, and D180 after transplantation were 1.1, 1.6, and 0.6 pg/ml, respectively. After D90 and D180 of transplantation, the IL-10 concentration was 1.3, 1.1, and 0.9 pg/ml, respectively. The concentration of MCP-1 was 600 pg/ml at baseline, 400 pg/ml 3 months after transplantation, and 300 pg/ml D180 after transplantation. D90 and D180 after transplantation, the EGF concentration was 210, 175, and 210 pg/ml, respectively. Therefore, patients’ anti-inflammatory cytokines were not significantly altered D180 after transplantation.

### The influence of stem cell transplantation on the ratio of proinflammatory to anti-inflammatory cytokines

Finally, the ratio of proinflammatory cytokines to anti-inflammatory cytokines will be used to assess the inflammation balance. In Table 4, the ratio from D-44 was compared to D90 and D180 after stem cell infusion. IL-4/IL-10 increased from 0.9 to 1.6 (*p* = 0.0035), IL-1/IL-10 decreased from 16.8 to 4.7 (*p* = 0.019), and IL-6/IL-10 decreased from 52.1 to 13.8 (*p* = 0.019) over the course of D90 compared to D-44. In addition, the ratios changed as follows: IL-2/IL-10 increased from 2.3 to 3.4% (*p* = 0.049), IL-6/IL-10 decreased from 52.1 to 16.1%, and IL-1/EGF median decreased from 0.1 to 0.1% (*p* = 0.004) over the course of D180 compared to D-44. Notably, the other ratio did not change significantly. Therefore, after stem cell transplantation, the ratio of proinflammatory to anti-inflammatory cytokines decreases in patients. Nonetheless, the ratios of IL-4/IL-10 at D90 and IL-2/IL-10 at D180 increased.

**Table 4:** Proinflammatory cytokine/anti-inflammatory cytokines ratios

	**D-44**	**D90**	***p*-value**		**D-44**	**D180**	***p*-value**
**IL-4/IL-10**			0.0035	**IL-2/IL-10**			0.0499
**Mean**	0.9	1.6		**Mean**	2.3	3.4	
**SD**	0.43	0.6		**SD**	0.65	1.71	
**IL-1β/IL-10**			0.0193	**IL-6/IL-10**			0.0262
**Mean**	16.8	4.7		**Mean**	52.1	16	
**SD**	17.39	5.42		**SD**	53.48	17.84	
**IL-6/IL-10**			0.0194	**IL-1β/EGF**			0.0043
**Mean**	52.1	13.8		**Mean**	0.1	0.1	
**SD**	53.48	14.83		**SD**	0.05	0.15	

Wilcoxon signed-rank test compared D90 and D180 to D-44 (baseline); *p*-value < 0.05 were considered statistically significant

*IL* Interleukine, *EGF* Epidermal growth factor

## Discussion

Age-related health problems are becoming increasingly associated with aging. The effects of aging are present in all human systems and physiological functions. The relation of age-related health problems is proposed to be caused by increased age in which there may be an imbalance in inflammation believed to play a significant role in the aging process. Therefore, inflammatory aging is an age-associated pathogenesis of numerous chronic diseases [22]. Inflammation aging intervention could provide new anti-aging and disease prevention strategies for the elderly [23]. Due to its abilities, such as self-renewal, differentiation, and cytokine secretion, stem cell therapy has found widespread application in regenerative medicine [24]. Stem cells are an internal factor of the body’s repair system. Therefore, stem cells have the potential to treat inflammation and prevent age-related chronic diseases.

The hypothesis of our study was to measure the efficacy of treating age-related inflammation and chronic disease through noninvasive, self-transplanted adipose tissue stem cells. After stem cell transplantation, the concentrations of proinflammatory cytokines, such as IL-1α, IL-1β, IL-6, IL-8, and TNF-α, in the blood of patients were significantly decreased, according to the study’s findings. Although anti-inflammation did not change significantly following stem cell infusion, the ratio of proinflammation cytokines to anti-inflammatory cytokines decreased. This means that stem cell transplantation improves chronic patients’ inflammatory balance.

The isolation of stem cells from adipose tissue is a less invasive procedure. In addition, self-transplantation of one’s own patient’s cells did not induce an immune response. The majority of clinical stem cell transplantation studies concluded that stem cells are safe. To date, there have been thousands of various clinical trials using stem cell therapy in the treatment of multiple diseases, such as cardiovascular, skeletal, and immune disorder and digestive system diseases. Furthermore, the safety of stem cells has been confirmed in high-quality clinical practice trials [19], similar to the results of our safety study.

The systemic elevation of IL-6 and TNF-α has been identified as an inflamm-aging marker that is responsible for multiple age-related diseases [6]. Consistently, chronic smokers exhibit a significant increase in proinflammatory cytokines such as TNF-α, IL-1, IL-6, and IL-8 and a decrease in anti-inflammatory cytokines such as IL-10 [25]. Not only in elderly patients but also in normal healthy humans showed that the levels of IL-6 and TNF were greater in participants ≥ 65 years of age than < 65 years of age [26]. In vitro study found that the fibroblasts from elder humans have higher secretion of IL-6 and IL-8 compared to young humans when it infected with Cytomegalovirus or stimulated by lipopolysaccharide [27]. In a senescent body, there is an accumulation of old, misfolded proteins and endogenously damaged molecules from damaged cells which activates the immune system [7]. Despite the fact that senescent cells have a protective effect on cell development, proinflammatory cells contribute to inflammation imbalance in the elderly [11] as a result of this natural process. Moreover, a digestive microbiome disorder brought on by aging increases the risk of infection, which triggers systemic inflammation [28]. The high level of serum inflammatory cytokines in elderly patients was capable of inducing cancer cell (MCF-7) proliferation, which was specifically related to IL-6 and IL-8 levels [29]. Multiple age-related morbidities and mortality are associated with high cytokine levels [30, 31]. Anti-inflammaging, through regulating the balance of proinflammation cytokines and anti-inflammatory cytokines, is deemed a necessary and effective strategy for chronic disease patients.

Immune modulation is the most promising mechanism for MSCs in the treatment of disease [32]. In fact, stem cells express a sensor for injury signals, which induces the secretion of immune regulators such as inhibitory immune ligands, complement components, and anti-inflammatory cytokines [33]. In addition, MSCs modulate the immune response by directly interacting with immune cells such as monocytes/macrophages, dendritic cells, T cells, B cells, and natural killer cells [34]. Unfortunately, it was discovered in aged humans that stem cell characteristics, number, structure, activity, and functions change [35]. Consequently, stem cell defects were linked to the aging process [36]. The inflammation-aging phenotype of elderly stem cells was discovered in relation to this study [37, 38].

From this perspective, this study consisted of MSC isolation, expansion, and reintroduction to patients. As anticipated, the patients’ inflammatory balance improved following transplantation. Although there is no clinical report on the effect of stem cells on anti-inflammaging, more than a hundred studies have reported the efficacy of stem cells in multiple organ disorders related to immune activities, such as bowel diseases [13], graft versus host disease [39], sepsis [40], Crohn’s disease [12], and ulcerative colitis [41]. The effectiveness of MSC transplantation was consistent with immune modulation for patients, according to these studies [20].

This is a phase 1 clinical trial, so the sample size is small, and there are no placebo groups with which to compare the treatment’s efficacy. Additionally, stem cell quality, which was dependent on the subject condition, could not be controlled in the study. Last, the evaluation of quality of life varies depending on the patient. The safety profile and initial efficacy of this therapy for anti-aging inflammation support future large-scale studies, control trial, and investigations into the underlying mechanism.

## Conclusion

The transplantation of AD-MSCs in aging-related low-grade inflammation patients was safe. Two doses of cell infusion reduced the patient’s inflammatory cytokines for the next 90 days.

### Supplementary Information

**Supplementary Material 1.**

## Abbreviations

AEs: Adverse events
D: Days
EGF: Epidermal growth factor
IL: Interleukin
IFN-γ: Interferon
MSCs: Mesenchymal stem cells
MCP-1: Monocyte chemoattractant protein-1
MS: Multiple sclerosis
RA: Rheumatoid arthritis
SAEs: Serious adverse events
TNFα: Tumor necrosis factor-alpha
VEGF: Vascular endothelial growth factor
